# Supplementary material for: A Likelihood Approach for Real-Time Calibration of Stochastic Compartmental Epidemic Models
Source: PLoS Comput Biol. 2017 Jan 17;13(1):e1005257. doi: 10.1371/journal.pcbi.1005257 (PMC5240920; doi:10.1371/journal.pcbi.1005257)
Supplement: S1 File — (TAR.GZ) [file pcbi.1005257.s014.tar.gz › HSPH_Online-SI-Revision/PF08/n100-mild/n100-mild_PF_table0.pdf]

1	R0	{1.24125, 3.38982}	{1.95676, 18.152}	{{1.90725, 17.2039}, {1.99041, 18.8124}}	{57.6441, 435.486}	inf	1822.	13203.1	{12408.3, 13754.5}	624.649	0.859595	newly	3621.	5499.54	{5119., 5746.}	51.879	0.181133	Reff	0.993708	1.44932	{1.42176, 1.47318}	45.8495	ar	34926.	93691.8	{92125., 94858.}	168.258
2	R0	{1.29554, 4.51878}	{1.94033, 17.6103}	{{1.79085, 15.4544}, {2.23737, 20.}}	{49.7702, 289.714}	inf	2837.	13608.1	{11307.1, 16586.3}	379.664	0.674953	newly	4247.	5645.94	{5376., 6080.}	32.9395	0.12313	Reff	0.958635	1.28109	{1.22717, 1.32577}	33.6366	ar	42828.	90315.2	{86146., 97427.}	110.879
3	R0	{1.2443, 3.64292}	{2.00872, 17.7904}	{{1.76542, 14.0853}, {2.21719, 20.}}	{61.4337, 388.356}	inf	2191.	14434.4	{11538.5, 16866.8}	558.805	0.813182	newly	4114.	6022.5	{5524., 6420.}	46.3904	0.164824	Reff	0.95617	1.35008	{1.26691, 1.40657}	41.1969	ar	37087.	90893.6	{84601., 95766.}	145.082
4	R0	{1.38465, 3.43331}	{2.99731, 18.3687}	{{3., 17.9981}, {3., 18.6654}}	{116.467, 435.014}	inf	3745.	27525.4	{27069.6, 27703.2}	634.99	0.866082	newly	6729.	10457.3	{10237., 10708.}	55.4064	0.191362	Reff	0.908206	1.09522	{1.07143, 1.11777}	20.5914	ar	49511.	104488.	{103634., 105353.}	111.041
5	R0	{1.25409, 4.1068}	{1.84118, 15.0099}	{{1.75172, 13.8043}, {2.13295, 20.}}	{46.8141, 265.489}	inf	2471.	12413.7	{10897.7, 16764.1}	402.376	0.696106	newly	3936.	6090.26	{5764., 6436.}	54.7322	0.189211	Reff	0.961511	1.26929	{1.22865, 1.38662}	32.01	ar	38571.	84407.5	{81927., 92998.}	118.837
6	R0	{1.28821, 4.2016}	{2.09185, 18.4194}	{{1.83568, 15.0872}, {2.33254, 20.}}	{62.3845, 338.391}	inf	2769.	15554.5	{12536.4, 18350.1}	461.738	0.744059	newly	4394.	6126.64	{5566., 6730.}	39.432	0.143318	Reff	0.936864	1.2564	{1.19724, 1.30608}	34.1075	ar	41468.	91800.3	{85819., 96993.}	121.376
7	R0	{1.24369, 2.56007}	{2.81784, 20.}	{{2.76453, 20.}, {2.88417, 20.}}	{126.571, 681.229}	inf	2411.	24970.6	{24101.1, 25979.}	935.696	1.01504	newly	6137.	9394.79	{9079., 9740.}	53.0844	0.18476	Reff	0.952269	1.53131	{1.50044, 1.56456}	60.8062	ar	38603.	97450.	{96685., 98296.}	152.441
8	R0	{1.232, 3.53942}	{1.93748, 18.0715}	{{1.83105, 16.2727}, {2.00644, 18.972}}	{57.2628, 410.578}	inf	1816.	15138.4	{13559.2, 16290.2}	733.614	0.920007	newly	3274.	6167.38	{5969., 6447.}	88.3745	0.274848	Reff	0.934361	1.31681	{1.28017, 1.33948}	40.9311	ar	34555.	94278.3	{91345., 96002.}	172.835
9	R0	{1.36262, 3.85631}	{2.67907, 19.2803}	{{2.40158, 17.3326}, {2.82914, 20.}}	{96.6119, 399.968}	inf	3694.	26066.4	{22258.3, 28288.8}	605.642	0.84697	newly	6191.	9618.14	{9167., 10006.}	55.3568	0.1911	Reff	0.926772	1.19547	{1.16891, 1.22506}	28.9933	ar	48410.	96207.	{93807., 98028.}	98.7338
10	R0	{1.28702, 4.67968}	{1.78709, 17.0468}	{{1.73108, 15.7568}, {1.83376, 18.3809}}	{38.8548, 264.272}	inf	2625.	11741.6	{10770.3, 12559.1}	347.3	0.649683	newly	3765.	4948.91	{4721., 5179.}	31.4452	0.118459	Reff	0.941571	1.16786	{1.11633, 1.19871}	24.0334	ar	41092.	79805.6	{77574., 81617.}	94.2119
11	R0	{1.35752, 3.47129}	{2.08158, 16.898}	{{2.07847, 16.8434}, {2.08427, 16.9575}}	{53.3373, 386.794}	inf	3108.	14787.3	{14725.1, 14865.1}	375.783	0.677405	newly	6127.	6568.97	{6463., 6682.}	7.21348	0.0302183	Reff	1.02022	1.39286	{1.38997, 1.39508}	36.5257	ar	44669.	87383.7	{87051., 87654.}	95.625
12	R0	{1.35929, 5.83647}	{1.8827, 17.5472}	{{1.83158, 16.4178}, {1.90971, 18.0478}}	{38.5064, 200.648}	inf	3507.	14108.	{12667.2, 14878.2}	302.282	0.60369	newly	4123.	5842.21	{5566., 6075.}	41.698	0.151146	Reff	0.975236	1.22852	{1.19543, 1.25162}	25.972	ar	46715.	89764.1	{87971., 91197.}	92.1526
13	R0	{1.28019, 3.29908}	{1.92686, 13.2352}	{{1.83121, 12.2603}, {1.94796, 13.5092}}	{50.5135, 301.177}	inf	2522.	14109.	{12864.4, 14423.1}	459.438	0.743472	newly	5146.	7809.84	{7454., 8182.}	51.7653	0.180801	Reff	0.960053	1.24476	{1.2133, 1.27767}	29.6552	ar	40941.	84391.3	{81910., 85421.}	106.129
14	R0	{1.36661, 3.48318}	{2.92545, 20.}	{{2.50742, 20.}, {3., 20.}}	{114.066, 474.188}	inf	3348.	26324.4	{24753.4, 26781.7}	686.273	0.895298	newly	5669.	9128.35	{8700., 9300.}	61.0222	0.206674	Reff	0.881791	1.04284	{1.01513, 1.11452}	18.2639	ar	48672.	100582.	{97937., 101604.}	106.654
15	R0	{1.35865, 3.84123}	{2.21164, 17.2334}	{{2.20762, 17.1741}, {2.21459, 17.332}}	{62.7824, 348.644}	inf	3969.	17480.2	{17361.4, 17562.3}	340.419	0.643862	newly	6860.	7535.77	{7396., 7661.}	9.85087	0.0407677	Reff	0.953229	1.36109	{1.35711, 1.36766}	42.7869	ar	48598.	89436.9	{89112., 89677.}	84.034
16	R0	{1.22879, 3.98329}	{1.7248, 17.3278}	{{1.65106, 15.8689}, {1.77323, 18.5154}}	{40.3657, 335.012}	inf	1615.	9794.47	{8779.74, 10601.9}	506.469	0.78121	newly	2786.	4173.69	{3928., 4371.}	49.8094	0.175166	Reff	0.98678	1.2885	{1.25033, 1.32056}	30.5761	ar	34405.	79349.	{76334., 81854.}	130.632
17	R0	{1.34925, 3.64524}	{2.67195, 19.5537}	{{2.52199, 20.}, {2.78941, 20.}}	{98.0326, 436.418}	inf	3518.	24549.2	{22265.3, 26705.1}	597.816	0.839124	newly	6327.	9212.29	{8291., 9751.}	45.6028	0.162417	Reff	0.979043	1.37259	{1.34582, 1.41413}	40.1974	ar	46871.	95974.2	{94188., 98047.}	104.762
18	R0	{1.33054, 2.25275}	{3., 20.}	{{3., 20.}, {3., 20.}}	{125.472, 787.804}	inf	3041.	27233.6	{27054.7, 27443.7}	795.548	0.95207	newly	8646.	10408.5	{10287., 10532.}	20.3856	0.0805429	Reff	0.969272	1.65121	{1.64496, 1.6579}	70.3562	ar	44336.	100903.	{100443., 101352.}	127.587
19	R0	{1.28394, 3.69859}	{2.21589, 19.936}	{{2.21092, 20.}, {2.24621, 20.}}	{72.5853, 439.017}	inf	2442.	15915.5	{15748.8, 16377.3}	551.739	0.813521	newly	4445.	5836.16	{5717., 6019.}	31.2972	0.118007	Reff	0.980378	1.3105	{1.29637, 1.32003}	33.6731	ar	39558.	93152.3	{92851., 93979.}	135.483
20	R0	{1.2883, 2.26493}	{3., 20.}	{{3., 20.}, {3., 20.}}	{132.865, 783.029}	inf	2636.	26976.8	{26303.6, 27415.8}	923.398	1.00999	newly	7800.	10540.7	{10325., 10713.}	35.1367	0.130725	Reff	1.01925	1.79212	{1.7656, 1.82415}	75.8274	ar	40768.	106955.	{106213., 107648.}	162.349
21	R0	{1.34098, 2.78621}	{2.80769, 18.0267}	{{2.3019, 13.0989}, {3., 20.}}	{109.376, 546.998}	inf	3142.	27745.8	{20510.2, 31361.7}	783.062	0.933057	newly	7245.	11341.9	{10778., 11641.}	56.5485	0.194432	Reff	0.972707	1.40798	{1.33682, 1.46317}	44.7489	ar	44605.	99168.3	{93724., 102352.}	122.326
22	R0	{1.33783, 5.95911}	{1.7842, 17.5911}	{{1.69487, 15.4803}, {1.85569, 20.}}	{33.3655, 195.196}	inf	3350.	12073.	{10642.5, 13175.7}	260.387	0.554977	newly	3826.	4983.42	{4738., 5240.}	30.2514	0.114426	Reff	0.988844	1.20853	{1.1696, 1.25523}	22.2166	ar	45418.	83026.1	{79354., 86542.}	82.8044
23	R0	{1.33359, 3.3069}	{2.22436, 16.5627}	{{1.94891, 13.1802}, {2.43705, 19.1088}}	{66.7949, 400.854}	inf	3385.	17298.6	{13646.8, 20069.}	411.037	0.701595	newly	6752.	7892.18	{7620., 8149.}	16.8866	0.0676093	Reff	0.971427	1.44553	{1.33352, 1.52567}	48.8053	ar	46245.	91527.6	{86095., 95444.}	97.919
24	R0	{1.30588, 2.56123}	{2.9275, 19.3327}	{{2.96614, 20.}, {3., 20.}}	{124.178, 654.823}	inf	2762.	31040.7	{30711.8, 32866.3}	1023.85	1.04408	newly	6606.	11632.7	{11323., 11902.}	76.0922	0.245651	Reff	0.932189	1.3374	{1.28393, 1.4158}	43.4691	ar	42383.	104107.	{104186., 106085.}	145.634
25	R0	{1.29813, 4.51849}	{1.96204, 20.}	{{1.93975, 20.}, {1.98077, 20.}}	{51.1436, 342.626}	inf	3099.	12881.1	{12426.3, 13261.1}	315.652	0.6186	newly	4627.	4766.11	{4587., 4933.}	3.35271	0.0142368	Reff	0.990123	1.35642	{1.34571, 1.36585}	36.9946	ar	43371.	84849.5	{84090., 85634.}	95.6364
26	R0	{1.33231, 3.99953}	{2.25954, 20.}	{{2.20018, 20.}, {2.33326, 20.}}	{69.5955, 400.059}	inf	3222.	18692.	{17765.6, 19486.2}	480.138	0.76323	newly	5352.	6834.13	{6448., 7154.}	27.693	0.105845	Reff	0.96366	1.31801	{1.28931, 1.33931}	36.7714	ar	44733.	96080.5	{94606., 97717.}	114.787
27	R0	{1.30555, 4.61445}	{1.96324, 20.}	{{1.94799, 20.}, {1.97181, 20.}}	{50.3764, 333.421}	inf	3092.	13451.1	{13030.7, 13747.}	335.028	0.638418	newly	4581.	4961.02	{4825., 5078.}	8.29557	0.0345185	Reff	0.983993	1.32998	{1.31871, 1.3487}	35.1617	ar	42624.	85598.5	{85044., 86183.}	100.822
28	R0	{1.26819, 5.34969}	{1.83807, 17.5016}	{{1.78424, 16.1016}, {1.94986, 20.}}	{44.9363, 227.151}	inf	2467.	11502.5	{10151.9, 13036.4}	366.254	0.666814	newly	3182.	4854.27	{4547., 5157.}	52.5541	0.182893	Reff	0.987438	1.30724	{1.26781, 1.34989}	32.3873	ar	38946.	85612.5	{83043., 89810.}	119.824
29	R0	{1.19925, 3.00394}	{1.94336, 17.2995}	{{1.85376, 16.1012}, {2.11283, 20.}}	{62.0482, 475.894}	inf	1551.	11601.1	{10354.6, 14461.3}	647.978	0.871018	newly	3362.	5064.18	{4775., 5491.}	50.63	0.177211	Reff	0.975746	1.42254	{1.37879, 1.48951}	45.7904	ar	31727.	89306.3	{86290., 94881.}	181.484
30	R0	{1.35749, 3.75483}	{2.11393, 13.8377}	{{2.06355, 13.6171}, {2.1691, 14.012}}	{55.7232, 268.53}	inf	3853.	19165.3	{18440.2, 19726.}	397.412	0.696581	newly	6557.	10131.5	{9768., 10487.}	54.5143	0.188808	Reff	0.934306	1.26263	{1.24951, 1.27665}	35.1408	ar	48244.	97013.5	{96042., 97933.}	101.089
31	R0	{1.27131, 4.2161}	{2.21274, 19.8556}	{{2.12911, 20.}, {2.28992, 20.}}	{74.0518, 370.947}	inf	2579.	15832.9	{15415.3, 16591.}	513.916	0.787345	newly	4228.	5940.91	{5702., 6213.}	40.5135	0.147447	Reff	0.992664	1.41004	{1.37804, 1.45141}	42.0463	ar	38299.	95361.7	{94131., 97013.}	148.993
32	R0	{1.31827, 6.18119}	{1.83746, 17.6124}	{{1.79928, 16.7701}, {1.87084, 18.2325}}	{39.3839, 184.935}	inf	3314.	12878.6	{11710.8, 13681.}	288.611	0.588615	newly	3614.	5299.95	{5041., 5555.}	46.6505	0.165995	Reff	0.960834	1.21312	{1.1848, 1.24278}	26.2572	ar	45262.	86805.7	{85306., 88121.}	91.785
33	R0	{1.20398, 1.35167}	{3., 20.}	{{3., 20.}, {3., 20.}}	{149.174, 1379.65}	inf	1581.	24315.6	{24132.3, 24484.2}	1437.99	1.18695	newly	7272.	9735.86	{9582., 9880.}	33.8815	0.126692	Reff	0.95218	1.91928	{1.9086, 1.92741}	101.567	ar	33329.	103920.	{103366., 104471.}	211.8
34	R0	{1.32776, 3.146}	{2.79491, 20.}	{{2.74723, 20.}, {2.82695, 20.}}	{110.498, 535.728}	inf	3143.	27505.7	{27133.8, 28020.3}	775.14	0.942029	newly	6043.	9803.1	{9625., 9994.}	62.2224	0.210062	Reff	0.90054	1.20778	{1.17417, 1.22819}	34.1174	ar	44872.	96885.1	{96151., 97528.}	115.914
35	R0	{1.279, 1.11336}	{3., 20.}	{{3., 20.}, {3., 20.}}	{134.558, 1696.36}	inf	1968.	19741.3	{19604.8, 19840.7}	903.116	1.00135	newly	7436.	8217.39	{8090., 8337.}	10.5082	0.0433681	Reff	0.863977	2.13169	{2.12675, 2.13563}	146.73	ar	41033.	102285.	{101961., 102601.}	149.275
36	R0	{1.20029, 1.94717}	{3., 20.}	{{3., 20.}, {3., 20.}}	{149.94, 927.132}	inf	1687.	23954.3	{23571., 24308.5}	1319.93	1.15224	newly	5772.	9398.16	{9211., 9542.}	62.8233	0.211678	Reff	0.983182	1.79817	{1.77909, 1.81645}	82.8932	ar	33234.	98785.6	{98273., 99158.}	197.243
37	R0	{1.33216, 3.69685}	{2.12267, 17.5133}	{{2.08918, 17.1305}, {2.14752, 17.7983}}	{59.3407, 373.736}	inf	3070.	17138.	{16625.9, 17569.3}	458.24	0.746726	newly	5464.	7139.21	{6923., 7316.}	30.659	0.116047	Reff	0.949151	1.26243	{1.25082, 1.27476}	33.0063	ar	45235.	88498.	{87821., 89057.}	95.6406
38	R0	{1.25434, 2.04314}	{3., 20.}	{{3., 20.}, {3., 20.}}	{139.17, 878.885}	inf	2420.	26240.9	{25832.4, 26803.3}	984.335	1.03513	newly	7800.	10166.7	{9975., 10324.}	30.3419	0.115042	Reff	0.972916	1.73659	{1.6982, 1.75369}	78.4929	ar	38662.	100851.	{100405., 101206.}	160.854
39	R0	{1.27466, 3.0741}	{2.46872, 17.8531}	{{2.26655, 15.4261}, {2.58, 19.0518}}	{93.6766, 480.76}	inf	2301.	24297.7	{20701.3, 26397.6}	955.962	1.02177	newly	4560.	9803.26	{9573., 10072.}	114.984	0.332328	Reff	0.912975	1.24388	{1.20357, 1.27167}	36.2444	ar	40181.	101290.	{98233., 103018.}	152.085
40	R0	{1.33045, 2.68154}	{3., 20.}	{{3., 20.}, {3., 20.}}	{125.488, 645.84}	inf	3224.	32211.4	{31941.7, 32429.1}	899.111	0.999606	newly	6981.	11562.8	{11425., 11688.}	65.6324	0.219128	Reff	0.885574	1.28711	{1.27842, 1.29321}	45.3422	ar	45951.	104339.	{103471., 105330.}	127.065
41	R0	{1.37003, 2.83984}	{3., 20.}	{{3., 20.}, {3., 20.}}	{118.973, 604.265}	inf	4061.	28945.5	{28769.6, 29053.6}	612.767	0.852945	newly	8819.	10842.1	{10717., 10968.}	22.9402	0.0896742	Reff	0.953239	1.53736	{1.53277, 1.54637}	61.2772	ar	48926.	101426.	{101167., 101687.}	107.305
42	R0	{1.3003, 2.94885}	{2.96028, 19.956}	{{2.92292, 20.}, {3., 20.}}	{127.661, 576.739}	inf	3021.	28938.2	{28250.4, 29634.2}	857.901	0.981068	newly	6589.	10536.3	{10261., 10734.}	59.9079	0.203775	Reff	0.931561	1.34632	{1.31916, 1.37769}	44.5235	ar	44237.	99593.2	{98739., 100344.}	125.136
43	R0	{1.33756, 2.92653}	{3., 20.}	{{3., 20.}, {3., 20.}}	{124.289, 583.403}	inf	3675.	27932.2	{26024.3, 28540.5}	660.059	0.880673	newly	8332.	10586.4	{9920., 10868.}	27.0572	0.103805	Reff	0.990757	1.59595	{1.54344, 1.62556}	61.0838	ar	47342.	101517.	{100968., 102035.}	114.434
44	R0	{1.35801, 2.92276}	{3., 20.}	{{3., 20.}, {3., 20.}}	{120.911, 584.285}	inf	4090.	31085.8	{30760.2, 31408.9}	660.044	0.880826	newly	8641.	11438.7	{11289., 11591.}	32.3774	0.121791	Reff	0.933387	1.43134	{1.42039, 1.44263}	53.3493	ar	47940.	104951.	{104372., 105611.}	118.921
45	R0	{1.31342, 4.02649}	{1.90157, 14.1096}	{{1.76259, 12.0559}, {2.25863, 20.}}	{44.7803, 250.42}	inf	3015.	13213.4	{10953., 18457.3}	338.254	0.633989	newly	4999.	6870.77	{6527., 7226.}	37.4429	0.137734	Reff	0.979207	1.24176	{1.18979, 1.35984}	26.8127	ar	42995.	82150.	{78293., 92024.}	91.0687
46	R0	{1.37919, 3.75889}	{2.87642, 20.}	{{2.83937, 20.}, {2.91181, 20.}}	{108.558, 432.072}	inf	3990.	26971.6	{26198.7, 27589.}	575.98	0.829858	newly	7235.	10003.	{9783., 10246.}	38.2582	0.140616	Reff	1.00539	1.45451	{1.42588, 1.47804}	44.6714	ar	49157.	98035.9	{97643., 98486.}	99.4342
47	R0	{1.37458, 3.56117}	{2.86416, 20.}	{{2.7948, 20.}, {2.91546, 20.}}	{108.366, 461.613}	inf	4168.	27745.4	{26725.6, 28481.4}	565.676	0.823122	newly	7293.	10054.2	{9692., 10330.}	37.8611	0.139312	Reff	0.937725	1.31959	{1.2874, 1.35473}	40.7225	ar	48478.	99143.	{97865., 100111.}	104.511
48	R0	{1.33393, 3.78296}	{1.99526, 20.}	{{1.99222, 20.}, {1.99764, 20.}}	{49.5778, 428.687}	inf	3354.	12736.	{12667.3, 12790.1}	279.725	0.579463	newly	5927.	4757.03	{4669., 4845.}	19.7397	0.0955464	Reff	0.973742	1.41996	{1.41847, 1.42213}	45.8255	ar	44885.	85869.6	{85468., 86240.}	91.3103
49	R0	{1.24918, 2.39046}	{2.43701, 18.1386}	{{2.11825, 14.0894}, {2.60012, 20.}}	{95.089, 658.789}	inf	1875.	20602.5	{15905.1, 23163.}	998.799	1.03543	newly	4978.	8487.7	{8064., 8830.}	70.5042	0.231483	Reff	0.958921	1.4416	{1.33998, 1.51423}	50.3353	ar	35969.	94841.	{89488., 97460.}	163.674
50	R0	{1.28281, 2.3032}	{2.98531, 18.645}	{{2.95167, 18.1087}, {3., 20.}}	{132.716, 709.528}	inf	2262.	25726.5	{24172.1, 29685.6}	1037.33	1.05445	newly	6004.	9939.07	{9503., 10746.}	65.5408	0.218474	Reff	0.938273	1.28023	{1.2292, 1.32859}	36.4454	ar	40223.	99182.9	{98503., 100130.}	146.583
